# How has peritoneal dialysis changed over the last 30 years: experience of the Verona dialysis center

**DOI:** 10.1186/s12882-015-0051-3

**Published:** 2015-04-14

**Authors:** Gianluigi Zaza, Carlo Rugiu, Alessandra Trubian, Simona Granata, Albino Poli, Antonio Lupo

**Affiliations:** Renal Unit, Department of Medicine, University-Hospital of Verona, Piazzale A. Stefani 1, 37126 Verona, VR Italy; Department of Public Health and Community Medicine, University of Verona, Verona, Italy

**Keywords:** Continuous Ambulatory Peritoneal Dialysis, Automated peritoneal dialysis, Patient survival, Clinical outcomes

## Abstract

**Background:**

The last decade has witnessed considerable improvement in dialysis technology and changes in clinical management of patients in peritoneal dialysis (PD) with a significant impact on long term clinical outcomes. However, the identification of factors involved in this process is still not complete.

**Methods:**

Therefore, to assess this objective, we retrospectively analyzed clinical records of 260 adult patients who started PD treatment from 1983 to 2012 in our renal unit. For the analysis, we divided them into three groups according to the time of starting dialysis: GROUP A (n: 62, 1983–1992), GROUP B (n: 66, 1993–2002) and GROUP C (n: 132, 2003 to 2012).

**Results:**

Statistical analysis revealed that patients included in the GROUP C showed a reduction in mean patients’ age (p = 0.03), smoking habit (p = 0.001), mean systolic blood pressure (p < 0.0001) and an increment in hemoglobin levels (p < 0.0001) and residual diuresis (p = 0.016) compared to the other two study groups. Additionally, patients included in GROUP C, mainly treated with automated peritoneal dialysis, showed a reduced risk of all-causes mortality and a decreased risk to develop acute myocardial infarction and cerebrovascular disease. Patients’ age, diabetes mellitus and smoking habit were all positively associated with a significant increased risk of mortality in our PD patients, while serum albumin levels and residual diuresis were negatively correlated.

**Conclusions:**

Therefore, the present study, revealed that in the last decade there has been a growth of our PD program with a concomitant modification of our patients’ characteristics. These changes, together with the evident technical advances, have caused a significant improvement of patients’ survival and a decrement of the rate of hospitalization. Moreover, it reveals that our pre-dialysis care, modifying the above-mentioned factors, has been a major cause of these clinical improvements.

**Electronic supplementary material:**

The online version of this article (doi:10.1186/s12882-015-0051-3) contains supplementary material, which is available to authorized users.

## Background

Chronic kidney disease (CKD) is a progressive and irreversible deterioration of kidney function classified by the last international guidelines into five stages according to glomerular filtration rate [1]. In the last phase (end stage renal disease, ESRD) the kidney impairment is advanced and cellular/metabolic functions are significantly altered and enable to guarantee normal body homeostasis. Consequently, at this stage, renal replacement therapies (RRTs, peritoneal- or hemo-dialysis) or renal transplantation are needed to ensure patient’s survival.

Although hemodialysis (HD) still represents the leading RRT, peritoneal dialysis (PD) procedure is utilized in more than 150,000 patients world-wide with a prevalence in Europe of 16% and in USA of 8% [2]. Recent data from the Italian Study Group of Peritoneal Dialysis have reported an incidence of this dialysis modality of approximately 20% with a prevalence of 15% [3,4].

PD seems to be a preferable choice for younger patients with high life expectancy and an elevated probability to undergo renal transplantation. In fact, this dialysis modality offers more flexibility allowing patients to continue working, a lesser cardiovascular impact and the maintenance of residual diuresis [5-7].

However, peritoneal catheter and dialysis solutions (characterized by high concentration of glucose, glucose degradation products, low pH and high osmolality) used to remove waste products generated from normal metabolic processes, uremic toxins and to normalize body fluid and electrolytes [8] may still determine the systemic activation of a complex intracellular machinery leading to inflammation and oxidative stress [9-11]. These conditions may induce systemic non-infectious clinical complications including cardiovascular diseases.

Furthermore, recent studies have identified several demographic factors (e.g., age > 75 years at the start of dialysis, BMI <18), clinical features (e.g., ischemic heart disease, anemia, heart failure and hemodynamic overload, cerebral vascular disease, peripheral vascular disease, diabetes) and metabolic causes (e.g., diabetes mellitus) associated with higher risk of mortality in PD patients compared with general population [12,13].

However, at the moment, only few reports have pointed out on the identification of risk factors for long-term clinical complications in PD patients living in Italy or other European countries [14-16]. It is unquestionable that geographically-related characteristics (e.g., diet, health care system), enhancing risk factors, may influence long term clinical outcomes.

Therefore, although monocentric, our study, performed on a large Italian cohort of PD patients (including 260 patients followed by our Renal/Dialysis Unit from 1983 to 2012), has been undertaken to identify changes across the last 30 years, to select clinical elements possibly predicting patients’ survival and to recognize targets of intervention useful to minimize the onset and development of severe dialysis-associated clinical complications.

## Methods

### Patients

In this study, we retrospectively analyzed clinical records of 260 adult patients (older than 18 years) who started PD at the Renal Unit of the Hospital-University of Verona from 1983 to 2012.

Patients with fewer than 6 months’ follow-up, who had been on HD or receiving a kidney graft before starting PD and had experienced at least one major cardiovascular event (myocardial infarction or cerebrovascular events, or who have undergone amputation) before dialysis were excluded.

Patients were, then, divided into 3 groups according to the time of starting PD:

- GROUP A (n: 62): patients starting PD from 1983 to 1992;
- GROUP B (n: 66): patients starting PD from 1993 to 2002;
- GROUP C (n: 132): patients starting PD from 2003 to 2012.

For all patients, we collected main demographic and clinical characteristics (Table 1). Estimated glomerular filtration rate (eGFR) has been calculated according to Cockcroft-Gault equation.

**Table 1:** **Trends in demographic and clinical characteristics among the three study periods**

	**GROUP A**	**GROUP B**	**GROUP C**	***p value***
	**1983-1992 (n = 62)**	**1993-2002 (n = 66)**	**2003-2012 (n =132)**	
**Follow-up months mean (SD)**	29.3 (25.1)	36.6 (27.4)	33.7 (26.9)	*0.298*
*Age years mean (SD)	63.5 (9.0)	59.7 (12.7)	59.0 (12.0)	*0.038*
**Gender**				
Males n. (%)	36 (58.1)	46 (69.7)	94 (71.2)	*0.174*
Females n. (%)	26 (41.9)	20 (30.3)	38 (28.8)	
**Smoking habits**				
No smokers n. (%)	51 (82.2)	39 (59.1)	77 (58.3)	*0.001*
Ex smokers n. (%)	7 (11.3)	11 (16.7)	38 (28.8)	
Smokers n. (%)	4 (6.5)	16 (24.2)	17 (12.9)	
*Diabetes				
No diabetes n. (%)	44 (71.0)	52 (78.8)	102 (77.3)	*0.532*
Diabetes n. (%)	18 (29.0)	14 (21.2)	30 (22.7)	
*Residual diuresis ml/die mean (SD)	777.8 (560.0)	924.2 (460.4)	997.7 (469.2)	*0.016*
*Systolic blood pressure mmHg mean (SD)	159.6 (25.2)	154.0 (19.9)	140.5 (15.4)	*<0.0001*
*Albumin g/dl mean (SD)	3.4 (0.49)	3.2 (0.5)	3.9 (2.8)	*0.06*
*Hb g/dl mean (SD)	8.7 (1.5)	9.6 (7.5)	11.7 (1.36)	*<0.0001*
*eGFR mean (SD)	6.1 (1.9)	6.4 (2.4)	6.9 (2.6)	*0.076*

eGFR: estimated Glomerular Filtration Rate. (*) At the start of PD treatment.

The study was carried out according to Declaration of Helsinki principles and approved by the Institutional Ethic Review Board of the University of Verona, Italy. All patients signed an informed consent.

### Statistical analysis

All data were expressed as frequencies, percentages and mean values or median according to the variables’ distribution. Parametric (*t*-test, ANOVA) and non-parametric (Mann–Whitney, Kruskal Wallis test) test and chi-square test/fisher test were used to assess differences in clinical and demographic features. Incidence rates of adverse events were calculated using the Poisson distribution and survival analysis was performed using Kaplan-Meier. Cox regression model was used to estimate the risk of acute myocardial infarction, cerebrovascular disease and vasculopathy. Tests have been corrected for baseline confounding factors as required. All tests were considered significant when p < 0.05. All statistical analysis were performed using SPSS, version 11.5.

## Results

### Demographic and clinical characteristics overtime

As showed in Table 1, we found significant differences in several demographic and clinical variables among the three study periods.

Statistical analysis revealed a significant and progressive reduction of the mean patients’ age (p = 0.03), smoking habit (p = 0.001), mean systolic blood pressure (p < 0.0001) across the study periods. On the contrary, hemoglobin levels (p < 0.0001) and residual diuresis (p = 0.016) were increasing overtime.

### Choice of PD methods

**Figure 1:**
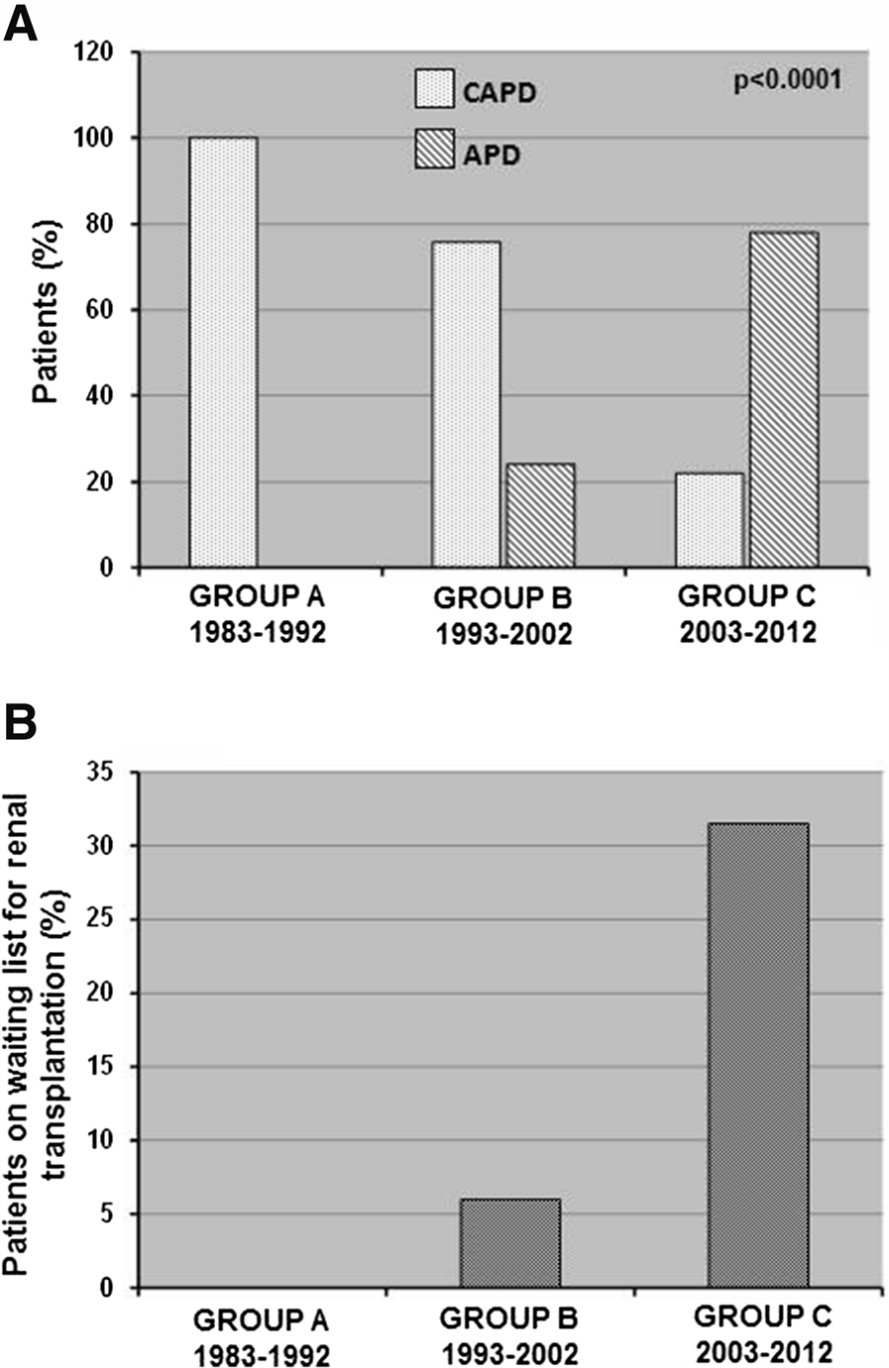
Patients’ distribution according to peritoneal dialysis (PD) modalities and percentage of patients on the waiting list for renal transplantation in the three study periods. The histograms represent **(A)** the percentage of patients on CAPD: Continuous Ambulatory Peritoneal Dialysis or APD: automated peritoneal dialysis and **(B)** the percentage of PD patients on the waiting list for renal transplantation in the three study groups. Group A: 1983–1992; Group B: 1993–2002; Group C: 2003–2012. P values calculated by fisher exact test.

From 1983 to 2012, we found a clear trend toward increased number of PD patients undergoing automated peritoneal dialysis (APD) (p < 0.0001). This could reflect a change of the demographic characteristics of our PD population (younger and still able to work) (Figure 1A).

### Waiting list for renal transplantation

During time, we found a significant increased number of PD patients on active renal transplant waiting list (p < 0.0001). This reflects a change of our center policy (mainly in the last decade) to use PD preferentially in younger patients without major comorbidities (e.g., cardio-vascular diseases, malignancies) (Figure 1B).

### All-causes mortality risk

**Figure 2:**
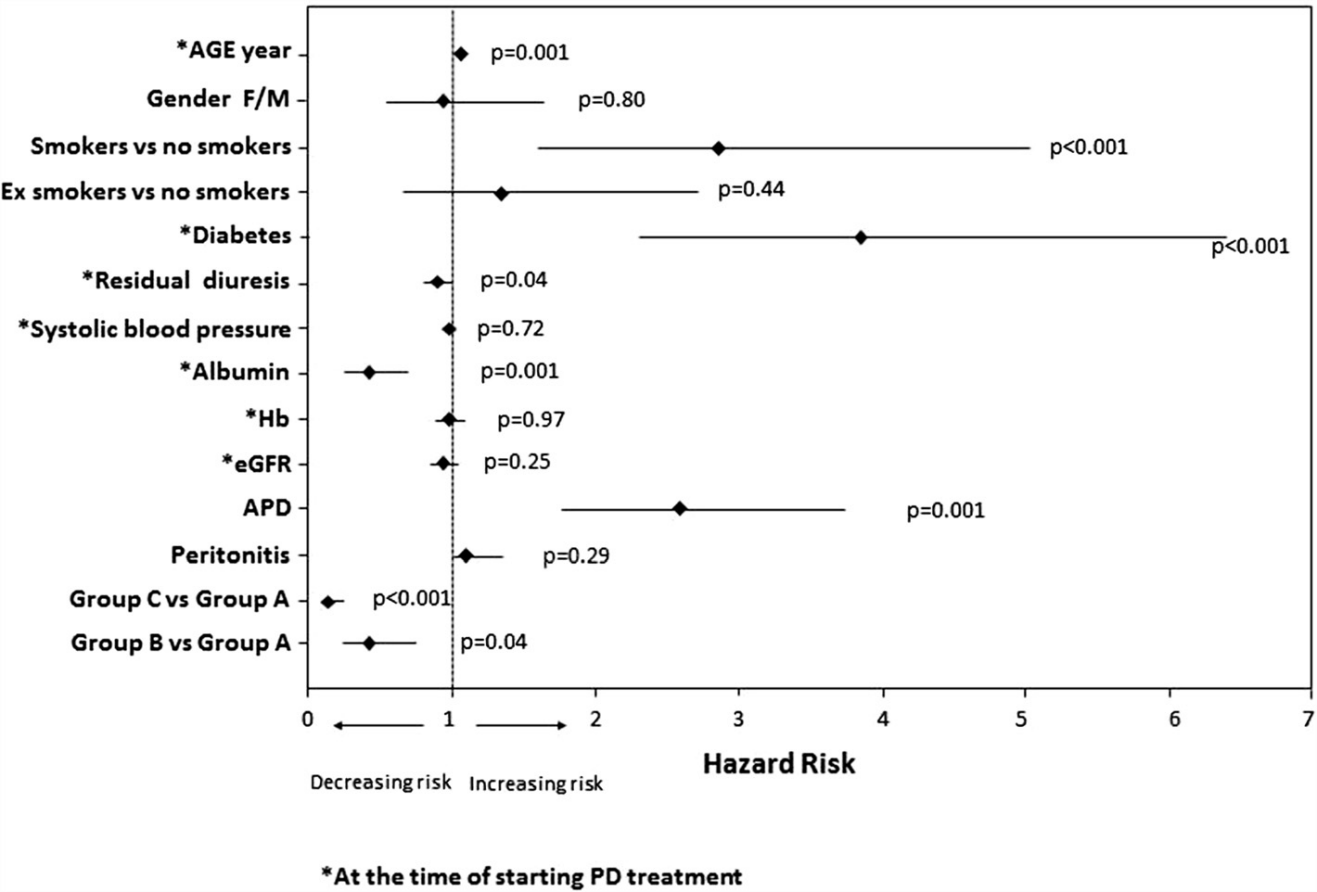
Multivariate Cox proportional hazard model for mortality according to several demographic and clinical characteristics. In this model are indicated Hazard ratio (HR) and 95% Coefficient interval (CI) for each factor analyzed. CVD: cardiovascular disease.

Multivariate analysis (Figure 2) showed that patients’ age (hazard ratio (HR): 1.1, p = 0.001), diabetes mellitus (HR: 3.9, p <0.001) and smoking habit (HR: 1.3, p <0.001) were all positively associated with an increased risk of all-causes mortality in our PD patients’ population, while serum albumin levels (HR: 0.6, p = 0.001) and residual diuresis (HR: 0.9, p = 0.04) were negatively correlated.

Gender, systolic blood pressure, hemoglobin levels and eGFR were not associated with patients’ survival.

**Figure 3:**
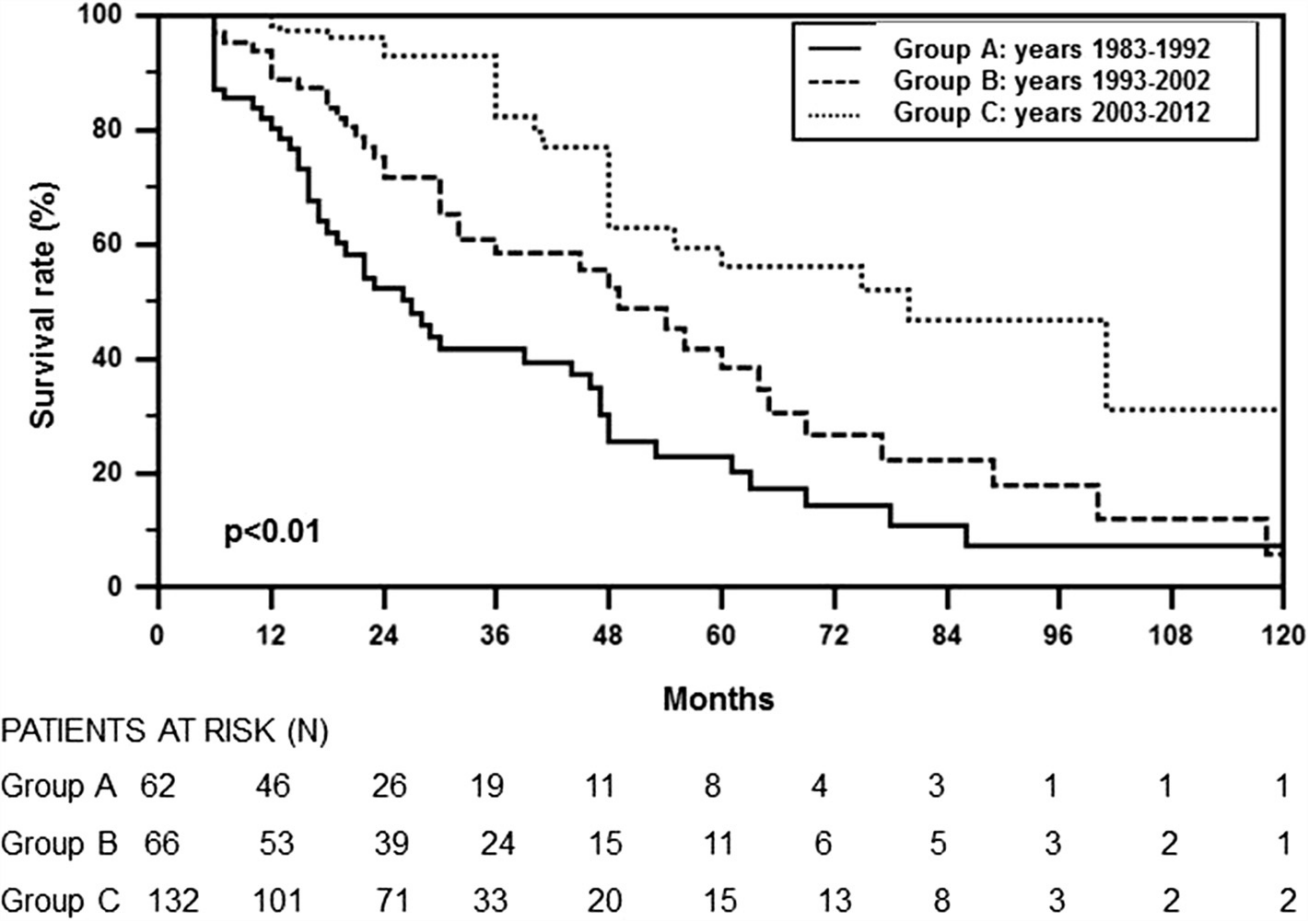
Survival rate in the three study groups by Kaplan-Meier. Survival rate of patients in the three study groups (Group A: 1983–1992, Group B: 1993–2002 and Group C: 2003–2012). Survival rate was better in Group C compared to the other two groups.

Kaplan-Meier revealed a better survival rate in PD patients of GROUP C compared to those included in the other two study-groups (Figure 3). Similar results were obtained evaluating transplantation and change in dialysis modality as competing outcomes and risks (Additional file 1: Table A and B, Figure S1). In particular, the statistical analysis confirmed a significantly higher risk of death in GROUP A *versus* both GROUP B and C (p < 0.0001) and an higher probability to undergo renal transplantation in last study period (GROUP C) compared to the other two periods (GROUP B and A) (p < 0.0001). On the contrary the risk to switch from PD to HD was similar among the three groups.

Interestingly, analyzing the trajectories of some of the above-mentioned clinical variables over-time (5 years), hemoglobin levels resulted constantly higher in GROUP C compared to B and A (Additional file 1: Table C). This could mainly be due to a complete correction of anemia (with a more rational use of ESAs) in the last 10 years.

### Risk of cardiovascular diseases

**Figure 4:**
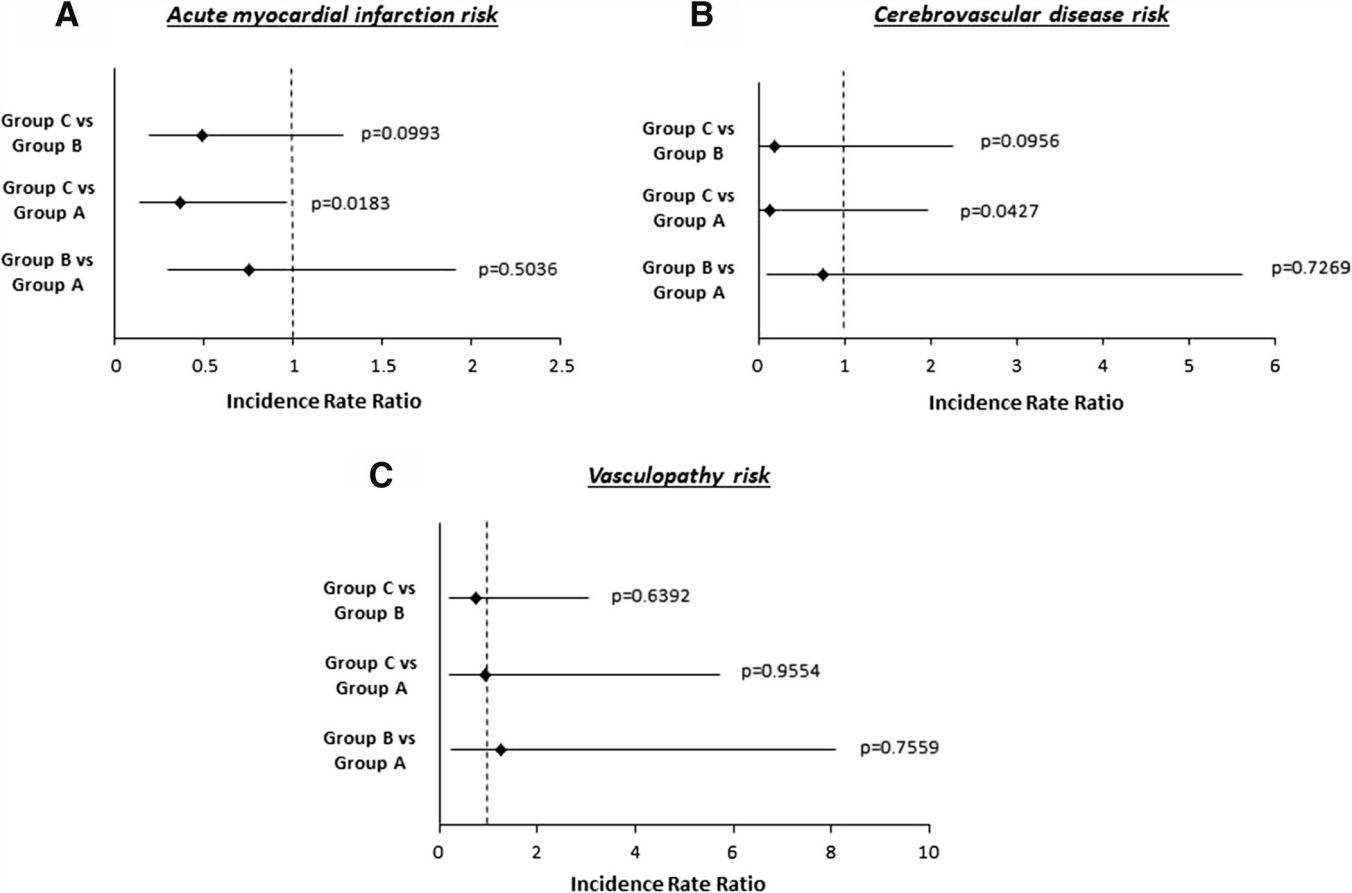
Incidence rate ratio of acute myocardial infarction **(A)**, cerebrovascular disease **(B)** and vasculopathy **(C)** according to the three study groups. Group A: 1983–1992; Group B: 1993–2002; Group C: 2003–2012. Figure has been built on the basis of Cox analysis.

As shown in Figure 4, there was a gradual reduction in the risk of developing acute myocardial infarction and cerebrovascular complications overtime.

We did not find any statistically significant difference in the risk of vascular disease.

### Days of hospitalization and complications

**Figure 5:**
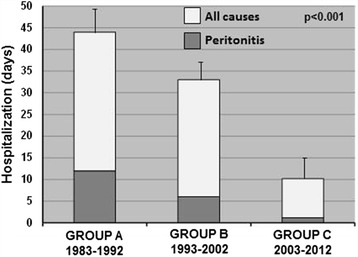
Days of hospitalization in the three study periods. The histograms represent the mean ± SD of the days of hospitalization for all causes (white bars) and peritonitis (gray bars) in each study group. Group A: 1983–1992; Group B: 1993–2002; Group C: 2003–2012.

Interestingly, patients included in GROUP C showed a lower period of overall hospitalization for all causes and peritonitis compared to Group B and A (Figure 5).

The major causative agents of peritonitis were *Staphylococcus Aureus* and *Epidermidis* followed by *Pseudomonas Aeruginosa* (Table 2). This condition was similar in the 3 study groups.

**Table 2:** **Frequency of occurrence of peritonitis and overall/post-peritonitis technique survival in the three study periods**

	**GROUP A (1983–1992)**	**GROUP B (1993–2002)**	**GROUP C (2003–2012)**
**Culture negative peritonitis (CNP)**	**35%**	**25%**	**17%**
**Culture positive peritonitis (CPP)**	**65%**	**75%**	**83%**
Staphylococcus aureus (% of total CPP)	20%	17%	19%
Staphylococcus epidermidis (% of total CPP)	40%	40%	36%
Pseudomonas (% of total CPP)	20%	18%	16%
Othergram-negativeorganisms (includingKlebsiella, Serratia and Enterobacter species) (% of total CPP)	15%	17%	18%
Other organisms (% of total CPP)	5%	8%	11%
**Post-peritonitis technique survival (at 1 year after peritonitis)**	**58%**	**75%**	**85%**
**Overall technique survival (at 5 years)**	**63%**	**73%**	**79%**

Technique and catheter survival after one episode of peritonitis were considerably higher in the last study period (GROUP C: 2003–2012) compared to the other two periods.

Overall technique survival was similar in the 3 groups. No catheter has been surgically removed and re-inserted after a peritonitis episode (according to Hospital policy).

## Discussion

Peritoneal dialysis (PD), although considered a valuable alternative renal replacement therapeutic option to hemodialysis (HD) in a great number of CKD patients (mainly young), is still associated with the development of severe long-term clinical complications (e.g., cardiovascular diseases) leading to significant reduction of patients’ survival [17].

Therefore, in the last decade, researchers and clinicians worldwide had work together to avoid or minimize these complications by introducing more “biocompatible” fluids (with more physiologic pH and reduced glucose degradation products) and by ameliorating the selection of eligible patients for this dialysis procedure [18,19].

Additionally, several strategies have been undertaken to minimize, particularly during the pre-dialysis follow-up period, all corrigible factors known to be associated with worst clinical outcomes of PD patients [20]. However, the complete identification of these elements represents a major target in nephrology.

To this purpose, we retrospectively analyzed our medical records regarding a large CKD patients cohort (n:260) starting PD treatment from 1983 to 2012.

Interestingly, several factors were significantly associated with an increased risk of mortality in our PD patients. Multivariate analysis showed that patients’ age, diabetes mellitus and smoking habit were all positively associated with an increased risk of all-causes mortality in our PD patients’ population.

The impact of age on survival is still debated. In fact, as a continuous home-based therapy, PD offers several potential advantages for older people, and it remains an important modality of renal replacement therapy, but patients with advanced age have an high risk to develop clinical complications and undergo peritonitis [21-24]

However, in a recent paper, Nessim et al. did not find any relationship between peritonitis and older age in the subgroup of patients who initiated dialysis in more recent years (2001 – 2005) [25] probably because of the recent advances in PD connection methods and exit-site care [26].

Also diabetes has been previously associated with worst outcomes in PD patients. Duong et al. have recently reported that a poor glycemic control (A1c ≥8% or serum glucose ≥300 mg/dl) appears to be associated with a decreased survival in PD patients. Authors suggested also that a better glycemic control could slow down the progression of microvascular disease and loss of residual renal function [27].

Therefore, to improve the management of diabetic patients undergoing PD represents a great challenge in nephrology.

To this purpose, icodextrin use has been encouraged in this large patients’ population. The benefit of this colloid osmotic agent, derived from maltodextrin, has been recently evaluated by Paniagua et al. in a prospective, randomized controlled trial in 60 diabetic patients undergoing PD [28]. These authors demonstrated that icodextrin, as compared with conventional glucose solution, reduces blood glucose concentration, a finding that was accompanied by a concomitant improvement in HbA1c and a reduction in insulin dosage. In addition, icodextrin-treated PD patients necessitated of a lower food intake.

Our study, then, reported that PD patients were highly vulnerable to the adverse consequences of smoking. This is in line with a paper published by Braatvedt et al. describing, in a large New Zealand database (more than 1000 patients), an higher age-adjusted mortality rate in PD patients with a history of current or former smoking compared to non-smokers [29].

On the contrary, serum albumin levels and residual diuresis were negatively associated with the risk of mortality for all-causes in our PD patients’ population.

In the past, serum albumin has been shown to predict all-cause mortality, and peritonitis risk in PD patients; however, the data are significantly more limited than for HD patients and most of the time based on small research studies [30-33].

The biological basis for the association of hypoalbuminemia with mortality remains uncertain. A main reason could be the magnitude of daily peritoneal protein losses (5–10 g/protein per day) in the dialysate effluent [34]. On the contrary, several researchers believe that since albumin is a negative acute phase reactant, this association in dialysis patients is secondary to the confounding influence of systemic inflammation [35]. A recent cross-sectional study of PD patients suggests that many of the patients with hypoalbuminemia are volume overloaded and hypervolemia may be an additional confounding influence [36].

Residual diuresis was the other clinical factor negatively associated with mortality in our PD patients confirming previous literature evidences reporting that a residual renal function (RRF) favoring the clearance of middle molecules, sodium removal and better control of volume status could have positive cardiovascular and systemic effects [37,38].

Moreover, as additional results of our study, we found a significant change of some of the above-mentioned mortality risk factors overtime. Mean patients’ age and smoking habit were reduced, while residual diuresis raised during-time.

We suppose that all these positive modifications have been possible thanks to the implementation in our Renal Unit of a specific pre-dialysis out-patients follow-up strategy by a dedicated multi-disciplinary team (involving medical doctors, nurses, psychologists, nutritionists) that, personalizing medical assistance, ensures an adequate metabolic balance, a good correction of anemia (with the more rational use of ESAs) and an implementation of all possible strategies to maintain the residual diuresis (for example by limiting the use of nephrotoxic drugs and by an overfluid control). Additionally, the correct education of our PD patients has increased their therapeutic compliance/adherence, reduced/stopped smoking habit and improved dietary intake.

In the last decade, then, this multi-disciplinary pre-dialysis program, together with the reduction of our patients’ age, has been responsible of the significant increment of PD patients undergoing APD modality (p = 0.0001). The APD, giving the possibility to continue to work and to maintain familial and social activities, can be considered a good “bridge” between CKD and renal transplantation (as showed in Figure 1A, at the moment, patients added to the waiting list for transplantation are more than 30%).

Other factors modified during the study periods were hemoglobin levels (increased) and systolic blood pressure (decreased). Both represent well known risk factors for cardiovascular mortality in the general and PD population [39,40].

This can partially justify the significant decreased risk of death for acute myocardial infarction and cerebrovascular complications in our PD patients overtime.

## Conclusions

Therefore, the present study, although limited by the exclusion of other important factors that could influence clinical outcomes (including nutritional status, peritoneal transport characteristics) and absence of a control group (i.e., patients undergoing hemodialysis treatment) clearly underlines that in the last decade there has been a significant increment in the number of patients undergoing PD and a profound change in their demographic and clinical characteristics.

Patients are younger, no smokers, with a residual diuresis, normal hemoglobin level and lower blood pressure. All these changes have definitely improved patients’ survival (all-causes and cardiovascular diseases) and caused a fall of the hospitalization rate. Moreover, our pre-dialysis care, modifying most of the above reported risk factors, has been a major actor of the clinical improvement observed in our PD patients’ population in the last 10 years.

## Additional file

Additional file 1:
**Additional Statistics.**

## Abbreviations

CKD: Chronic kidney disease
RRT: Renal replacement therapies
HD: Hemodialysis
PD: Peritoneal dialysis
BMI: Body mass index
eGFR: Estimated glomerular filtration rate
APD: Automated peritoneal dialysis
HR: Hazard ratio
RRF: Residual renal function
ESRD: End stage renal disease
