# Supplementary material for: How has peritoneal dialysis changed over the last 30 years: experience of the Verona dialysis center
Source: BMC Nephrol. 2015 Apr 14;16:53. doi: 10.1186/s12882-015-0051-3 (PMC4404116; doi:10.1186/s12882-015-0051-3)

**Supplemental information for:**

### HOW HAS PERITONEAL DIALYSIS CHANGED OVER THE LAST 30 YEARS:

**EXPERIENCE OF THE VERONA DIALYSIS CENTER.**

Gianluigi Zaza^1^, Carlo Rugiu^1^, Alessandra Trubian^1^, Simona Granata^1^, Albino Poli^2^,

Antonio Lupo^1^

^1^ Renal Unit, Department of Medicine, University-Hospital of Verona, 37124, Verona, Italy

^2^ Department of Public Health and Community Medicine, University of Verona, Verona, Italy

Gianluigi Zaza: [Gianluigi.zaza@univr.it](mailto:Gianluigi.zaza@univr.it)

Carlo Rugiu: [carlo.rugiu@ospedaleuniverona.it](mailto:carlo.rugiu@ospedaleuniverona.it)

Alessandra Trubian: [alessandra.trubian@yahoo.it](mailto:alessandra.trubian@yahoo.it)

Simona Granata: [simona.granata@univr.it](mailto:simona.granata@univr.it)

Albino Poli: [albino.poli@univr.it](mailto:albino.poli@univr.it)

Antonio Lupo: [antonio.lupo@univr.it](mailto:antonio.lupo@univr.it)

**Address correspondence to:**

Gianluigi Zaza, MD, PhD

Renal Unit, Department of Medicine,

University-Hospital of Verona, Verona, Italy.

Piazzale A. Stefani 1, 37126, Verona (VR), Italy

Tel. 045.8122528;

fax 045.8027311

E mail: [gianluigi.zaza@univr.it](mailto:gianluigi.zaza@univr.it)

**Table A. Estimates of the probability of death at different time-points.**

| **Outcome** | **Time points (months)** | | | | | |
| --- | --- | --- | --- | --- | --- | --- |
|  | 0 | 24 | 48 | 72 | 96 | 120 |
| Group A death | 0 | 0.43548 | 0.61290 | 0.67742 | 0.70968 | 0.70968 |
| Group B death | 0 | 0.25758 | 0.38225 | 0.49134 | 0.52251 | 0.55368 |
| Group C death | 0 | 0.05809 | 0.19019 | 0.21087 | 0.23571 | 0.25645 |
| Group A HD | 0 | 0.14516 | 0.19355 | 0.24194 | 0.25806 | 0.25806 |
| Group B HD | 0 | 0.09091 | 0.24545 | 0.26104 | 0.27662 | 0.27662 |
| Group C HD | 0 | 0.17340 | 0.28945 | 0.31082 | 0.33674 | 0.33674 |
| Group A Transplant | 0 | 0 | 0.01613 | 0.01613 | 0.01613 | 0.01613 |
| Group B Transplant | 0 | 0.06061 | 0.13853 | 0.15411 | 0.15411 | 0.15411 |
| Group C Transplant | 0 | 0.15598 | 0.31354 | 0.32388 | 0.36535 | 0.36535 |

**Table B. Test equality across groups.**

| **Outcome** | **Statistics** | **P value** | **df** |
| --- | --- | --- | --- |
| death | 51.11 | <0.0001 | 2 |
| HD | 1.32 | 0.52 | 2 |
| transplant | 27.65 | <0.0001 | 2 |

**Table C. Trajectories of clinical variables over-time (5 years).**

**Figure 1S. Probability of death at different time-points.**


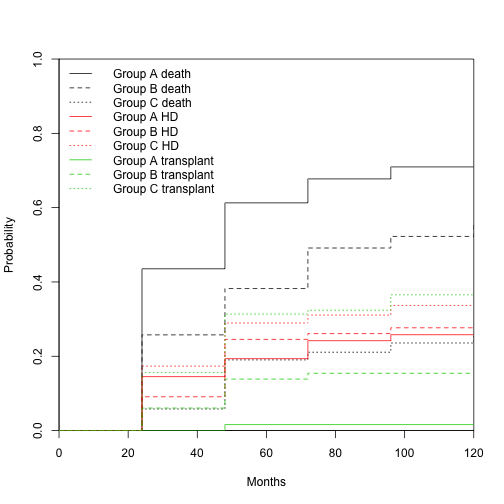

Supplement: Additional file 1: — Additional Statistics. [file 12882_2015_51_MOESM1_ESM.docx]
